# Complete genome sequences of bacteriophages Guetzie and SirVictor, isolated from *Microbacterium foliorum*

**DOI:** 10.1128/mra.01074-23

**Published:** 2024-01-31

**Authors:** Zefanias Ngove, Raegen Matthews, Jack Goedken, Sophia Huntington, Lauren Kirkle, Sean T. Coleman

**Affiliations:** 1 Biology Department, Wartburg College, Waverly, Iowa, USA; 2 College of Dentistry, University of Iowa, Iowa City, Iowa, USA; Loyola University Chicago, USA

**Keywords:** bacteriophages, genome analysis

## Abstract

Cluster EA4 Guetzie and SirVictor are lytic siphoviral bacteriophages that were isolated from soil in Waverly, Iowa, using *Microbacterium foliorum* NRRL B-24224 as the host. The Guetzie and SirVictor genomes are both 39,758 bp each, and both contain 58 predicted protein-coding genes with one tRNA gene each.

## ANNOUNCEMENT

Bacterial species of the genus *Microbacterium* are Gram-positive rod-shaped aerobic organisms that are found on plants, in soil, and in water and are of human interest due to their potential to cause opportunistic infections (1, 2). Isolating and characterizing bacteriophages that cause infection, *Microbacterium* offers insight into bacteriophage evolution and diversity and the development of phages as a therapeutic for controlling infections (3, 4). Here, we present the genome sequences of two bacteriophages that infect *Microbacterium foliorum* NRRL B-24224.

**Fig 1:**
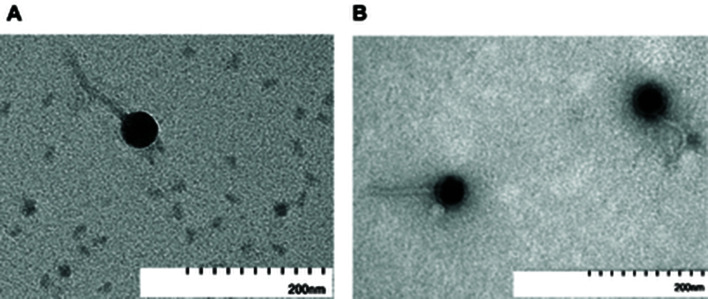
Transmission electron micrographs of Guetzie (**A**) and SirVictor (**B**) High-titer lysates were placed on Formvar-coated grids, negatively stained with Uranyless (5), and imaged at 60 kV and 100 kV, respectively. Guetzie has a tail length diameter of 136 to 141 nm and an isometric capsid of 55 to 64 nm in diameter (*n* = 3), and SirVictor has a tail length of 145 to 151 nm and an isometric capsid 48 to 55 nm in diameter (*n* = 3)

Bacteriophages Guetzie and SirVictor were isolated from semi-arid soil near Wartburg College, Waverly, Iowa, USA (Table 1), using standard protocols (5). Soil specimens were collected, subjected to a wash utilizing peptone-yeast extract-calcium (PYCa) liquid medium, and subsequently filtered using 0.22-µm filters to extract the bacteriophages from the soil. The filtrate was inoculated with *M. foliorum*, and the resulting mixture was plated in a soft agar overlay onto separate PYCa plates and incubated at 30°C for 24 hours. The bacteriophages successfully replicated during this incubation period, yielding small, discernible clear plaques. The bacteriophages were purified through three rounds of plating. Transmission electron microscopy using negative staining was conducted on purified lysate, revealing siphoviral morphologies for both bacteriophages. Guetzie and SirVictor had a tail length of 136 to 141 nm and an isometric capsid of 55 to 64 nm in diameter (*n* = 3) and a tail length of 145 to 151 nm and an isometric capsid of 48 to 55 nm in diameter (*n* = 3) respectively (Fig. 1).

**TABLE 1:** Characteristics of bacteriophages Guetzie and SirVictor

Phage name	GenBank accession no.	SRA accession no.	Location (GPS coordinates)	Genome size (bp)	Cluster	G+C content (%)	Genome ends	Average nucleotide % identity^*a*^	No. of CDSs^*b*^	No. of genes assigned a function	No. of tRNAs
**Guetzie**	OR253907	SRX22366552	42.730129 N, 92.482883 W	39,758	EA4	64.2	Circularly permuted	99.9	59	26	1 (Phe)
**SirVictor**	OR475284	SRX22366556	42.73104 N, 92.48409 W	39,758	EA4	64.2	Circularly permuted	99.9	59	26	1 (Phe)

^*a*^ Calculated using OrthoANI (6).

^*b*^ CDSs, coding DNA sequences.

DNA was isolated from both Guetzie and SirVictor lysates using the Promega Wizard DNA cleanup kit. The genome was sequenced using an Illumina MiSeq sequencer (v3) reagent after the library was prepared using the NEBNext Ultra II FS kit, yielding 3,754,519 and 537,197 single-end 150-bp reads for Guetzie (14,165-fold genome coverage) and SirVictor (2,026-fold coverage), respectively. Raw reads were assembled and checked for completeness using Newbler v2.9 with default parameters and Consed v29 as described previously (7). Information regarding GenBank/SRA numbers, GPS coordinates, and the genome information can be seen in Table 1. The G+C content for both Guetzie and SirVictor is similar to that of the host *M. foliorum* (68.7%) (8) and was assigned to cluster EA4 based on a gene content similarity of ≥35% to phages in the Actinobacteriophage Database (8, 9).

The genomes were annotated using DNA Master (http://cobamide2.bio.pitt.edu), PECAAN (https://blog.kbrinsgd.org), Glimmer v3.02 (10), GeneMark v2.5 (11), Starterator v1.1 (http://phages.wustl.edu/starterator), and Phamerator (12). Predicted gene functions were determined using BLASTp v2.9 (13), HHpred (14), TMHMM2 (https://services.healthtech.dtu.dk/service.php ?TMHMM-2.0), and SOSUI (15), and tRNAs were identified using ARAGORN v1.2.38 (16) and tRNAscan-SE v3.0 (17). Default settings were used for all programs. Annotation revealed 58 protein-coding genes and one tRNA in both genomes. The genomes of Guetzie and SirVictor showed 99.9% nucleotide identity via OrthoAni (6) including at least two nucleotide differences. There are eight EA subclusters (EA1-8). Interestingly, most EA3, EA4, EA5, and EA6 phages have a single tRNA-Ala (3) while Guetzie and SirVictor have a single tRNA-Phe.

## Data Availability

The sequencing results for Guetzie are available in GenBank with Accession No. OR253907 and Sequence Read Archive (SRA) Accession No. SRX22366552. Likewise, for SirVictor, the GenBank Accession No. is OR475284 and the SRA Accession No. is SRX22366556.
